# Effectiveness and Experience of Implementing Digital Interventions to Promote Smoking Cessation Among Adults With Severe Mental Illness: A Systematic Review and Meta-analysis

**DOI:** 10.1093/ntr/ntae237

**Published:** 2024-10-09

**Authors:** Lisa Huddlestone, Emily Shoesmith, Jodi Pervin, Rosie Stevens, Simon Gilbody, Elena Ratschen

**Affiliations:** Department of Health Sciences, University of York, Heslington, York, UK; Department of Health Sciences, University of York, Heslington, York, UK; Department of Health Sciences, University of York, Heslington, York, UK; Department of Health Sciences, University of York, Heslington, York, UK; Department of Health Sciences, University of York, Heslington, York, UK; Hull York Medical School, University of York, Heslington, York, UK; Department of Health Sciences, University of York, Heslington, York, UK

## Abstract

**Introduction:**

Digital technology is increasingly used to support interventions targeting smoking cessation in people with severe mental illness (SMI). However, little is known about their implementation and effectiveness in this population. We aimed to determine the effectiveness, stakeholder experiences, factors influencing implementation, and quality of reporting of digital interventions for smoking cessation in adults living with SMI.

**Methods:**

Five online bibliographic databases were searched for articles published between December 31, 2000 and January 31, 2023. Studies involving adults accessing treatment for alcohol and substance use disorders, neurocognitive disorders, and terminal illnesses were excluded. Risk of bias was assessed using the Mixed Methods Appraisal Tool. A Mantel–Haenszel random-effects meta-analysis of randomized controlled trials was conducted. Participant experience and intervention implementation were explored using a narrative synthesis. Quality of reporting of interventions was assessed using the Template for Intervention Description and Replication checklist.

**Results:**

Thirty-one studies enrolling 3794 participants were included. Meta-analysis of biochemically verified abstinence at longest follow-up (month 1 to month 6) did not find an overall effect in favour of intervention (log risk ratio = 0.66, 95% confidence interval = −0.005 to 1.37). Interventions tailored to people with SMI were perceived as acceptable. Implementation strategies concentrated on overcoming practical challenges at the participant/user level.

**Conclusions:**

No evidence of the effectiveness of digital interventions to support smoking cessation in people with SMI was found. The importance of tailoring interventions to the needs of people with SMI is highlighted. Robust reporting of implementation is required to enhance future efforts to support smoking cessation in adults with SMI.

**Implications:**

The findings of this review add to the emerging evidence on digital interventions to support smoking cessation among people with SMI. We highlight the importance of tailoring interventions to the population, particularly considering the role of mental illness and the side effects of psychotropic medication in the accessibility and usability of digital interventions.

## Introduction

Severe mental illnesses (SMIs) are defined by diagnosis, degree of disability, and the presence of some abnormal behavior.^1^ They include primarily psychotic disorders such as schizophrenia, schizoaffective disorder, and bipolar disorder, as well as major depressive disorder and some trauma and stress disorders.^2,3^ People with SMI experience increased rates of premature mortality and morbidity in comparison to those individuals without mental illnesses, with between 14 and 20 life-years lost, largely due to cardiovascular disease and as a result of modifiable risk factors such as smoking.^4^ Adults with mental health conditions are more likely to experience higher nicotine dependence, develop smoking-related illnesses, and long-term quit rates among this population are lower than those in individuals without a mental health condition.^5–7^ Although people with mental health conditions are just as motivated to quit,^5,8^ they are less likely to receive the support they require compared with smokers without mental health conditions.^9^

In recent years, there has been an expansion in the development and use of digital interventions to improve mental health and well-being.^10,11^ While many digital interventions are available through commercial app marketplaces, they are also increasingly being commissioned in health services, including within mental health settings, to supplement or enhance existing nondigital treatments and provide a means of addressing gaps in service provision.^12^ However, developing digital health technologies that individuals with SMI can access can be challenging because of illness-related factors such as cognitive impairments, limited digital literacy and skills, or access to Wi-Fi,^13,14^ as well as the side effects of psychotropic medications, which can cause drowsiness, fatigue, blurred vision, and difficulty reading.^15,16^ Given that, without adequate design considerations, the move toward increased digital delivery of interventions has the potential to amplify the inequality and poor outcomes already experienced by people with SMI.

Published evidence suggests that digitally delivered interventions may aid smoking cessation or reduce tobacco consumption in the general population,^17–19^ as well as having advantages over more traditional nondigital approaches, such as ease of accessibility, personalization of interventions with real-time feedback, scalability, and cost-effectiveness.^20^ However, such interventions were not developed for psychiatric populations and do not consider the specific barriers and enablers to quitting smoking experienced by people with SMI, including, for example, misconceptions of smoking being therapeutic, concerns about potential negative effects of quitting smoking on mental health,^21^ and the need for flexible and extended behavioral support to account for typically high levels of nicotine dependence that can render quitting challenging.^22,23^ In addition, previous reviews have highlighted that studies involving nonpsychiatric populations are of lower quality than average and called for greater uniformity in the conduct and reporting of trials.^20,24^ Previous systematic reviews on digital health interventions for people with SMI have concluded such interventions appear promising, indicating feasibility, effectiveness, and acceptability.^25,26^ While these reviews indicate the promise of digital interventions, they were narrative and did not attempt to estimate an overall/pooled effect of the interventions. Furthermore, scarce information exists on the strategies used to implement digital interventions within SMI populations and the participant experience of receiving the intervention. Therefore, the overarching aim of this review was to evaluate the current evidence on digital interventions for smoking cessation in adults living with SMI. Specifically, the objectives were to determine:

1. The effectiveness of digital interventions in supporting smoking cessation in adults with SMI.
2. Participant, caregiver, and/or staff experiences of digital interventions to support smoking cessation in adults with SMI.
3. Factors influencing implementing and delivering digital interventions for adults with SMI.
4. The quality of reporting of digital interventions to support smoking cessation in adults with SMI.

## Methods

The systematic review protocol was preregistered with the International Prospective Register of Systematic Reviews (PROSPERO); reference: CRD42022309963. The methodology is reported according to the Preferred Reporting Items for Systematic Reviews and Meta-analyses (PRISMA) guidelines.^27^

### Search Strategy

Searches were conducted for English language articles in five online bibliographic databases: MEDLINE, EMBASE, CINAHL, PsycInfo, and Applied Social Sciences Index and Abstract from December 31, 2000, when computers and innovative technologies became widely available to January 31, 2023. The search strategy included terms relating to the population, intervention, and outcomes (Table S1). An additional Google Scholar search using relevant keywords and phrases was conducted, and the first 10 pages of results were reviewed. Endnote X9 was used to record the number of retrieved articles from each database.

### Inclusion and Exclusion Criteria

Studies were identified for inclusion based on the population, intervention, comparator, and outcome method for eligibility. Randomized controlled trials (RCTs) (including feasibility and pilot trials), observational cohort studies, and surveys or qualitative studies were considered.

#### Population

Studies including adults (aged ≥ 18 years) with a diagnosis of SMI who smoke, defined here as schizophrenia, schizotypal, and delusional disorders (ICD-10^3^ codes F20–F29 or DSM equivalent^28^), mood disorders (ICD-10 codes F30–F39 or DSM equivalent), neurotic, stress-related, and somatoform disorders (ICD-10 codes F40–F48 or DSM equivalent), and disorders of adult personality and behavior (ICD-10 codes F60–F69 or DSM equivalent) were eligible for inclusion. Studies including health professionals and/or the carers and family members of adults with SMI who smoke were also eligible for inclusion. Studies were excluded if participants were accessing a treatment service for alcohol or substance use, a diagnosed neurocognitive disorder (eg, dementia or learning disability), or a terminal illness.

#### Intervention

Studies describing the development, implementation, or evaluation of digital interventions for smoking cessation delivered to adults with SMI were included. Interventions using pharmacotherapies or electronic cigarettes, or in-person support (either from caregivers or mental health professionals) in addition to digital interventions, were eligible for inclusion. Both bespoke designs and interventions adapted from generic designs to suit the needs of the SMI population were also considered.

#### Comparator

A control comparator was not necessary for inclusion in this review. Studies with or without the following controls were considered: no treatment control groups, waiting-list control, normal practice, or any other intervention described by the authors as a comparator.

#### Outcomes

Outcomes included RCTs (including randomized feasibility studies and pilot trials) and cohort studies that reported smoking abstinence, either via self-report and/or validated by biochemical verification at any timepoint post-discharge. Additional outcomes included stakeholder experiences of digital interventions, and factors influencing the implementation or delivery of digital interventions.

### Study Selection and Extraction

Retrieved studies were imported into the Covidence systematic review software web platform and screened for eligibility. Following the removal of duplicates, study titles and abstracts were independently screened for eligibility by two reviewers (LH and ES). Where disagreements arose as to the inclusion of a study, these were settled through discussion, and when consensus was not reached, a third reviewer (JP) was consulted. Two reviewers (ES and JP) independently undertook the full-text screening. In all cases where disagreements arose, these were settled through discussion with a third reviewer (LH). Data were extracted on study design, sample characteristics, setting, intervention, follow-up periods, and outcomes of interest using a bespoke data collection tool.

### Assessment of Risk of Bias

The included studies were assessed using the Mixed Methods Appraisal Tool (MMAT) (version 11).^29^ The MMAT allows for the critical appraisal of the methodological quality of RCTs, nonrandomized studies, quantitative descriptive studies, qualitative research, and studies utilizing mixed methods.^30^ Two authors (JP and RS) independently rated each study, and any differences of opinion were settled through discussion with a third author (ES). In the revised MMAT,^30^ using an overall numerical score is discouraged, with Hong and colleagues favoring a detailed presentation of the ratings against the MMAT criteria to reflect the quality of the included studies. Following the assessment of each study against the criteria, we compared the quality of included studies by contrasting their results to determine if a study was of high, good, fair, or poor quality.

### Data Analysis

To address the effectiveness of digital interventions in supporting smoking cessation in adults with SMI (objective 1), a Mantel–Haenszel meta-analysis was conducted for RCTs using Stata 18.^31^ Heterogeneity between study outcomes was assessed using the *I*^2^ statistic, suitable for smaller meta-analyses.^32^ Due to the likelihood of significant heterogeneity, a random-effects model was used. Post hoc subgroup analyses to separate studies by those with digital control groups and those with nondigital control groups were also conducted. For all meta-analyses, participants lost to follow-up were treated as nonabstinent, except those who were reported as deceased.^33^ Publication bias was assessed using funnel plots. Where visual inspection indicated potential funnel plot asymmetry, Egger’s regression intercept, used to quantify publication bias,^34^ was used to investigate this.

To address participant, caregiver, and/or staff experiences of digital interventions and factors influencing the implementation and delivery of these interventions (objectives 2 and 3), a narrative synthesis was conducted to synthesize qualitative and quantitative findings related to acceptability and usability, and factors perceived to influence implementation and delivery.

To evaluate the quality of reporting of the digital interventions (objective 4), the Template for Intervention Description and Replication (TIDieR) checklist was used.^35^ Data from each published article and any supplementary material referred to in the reporting were assessed. Two authors’ (LH and JP) evaluated each study using a customized Excel spreadsheet. As recommended in the guidance issued by the TIDieR committee, completion of the checklist followed the guide, and each item was assessed based on the explanation and elaboration it provides.^35^ Each item was scored “yes,” “no,” or “not applicable.” Items that fully met the criteria were scored “yes,” while those missing or only partially satisfying the criteria were rated “no.” The notation of “not applicable” was used where the intervention was not personalized/modified or in cases where fidelity or adherence was not assessed.

Owing to the variety of study designs, only the description of intervention conditions was evaluated. Proportions and 95% confidence intervals (CIs) were calculated for articles rated “yes” on each TIDieR item. Cohen’s kappa (*k*) was calculated to assess the agreement between reviewers overall and by TIDieR checklist item. Interpretation of the coefficient is described as^34^: poor = <0; slight = 0.00–0.20; fair = 0.21–0.40; moderate = 0.41–0.60; substantial = 0.61–0.80; and almost perfect = 0.81–1.0. All data were analyzed in IBM SPSS v28.^36^

## Results

### Risk of Bias

Overall, the quality of studies was found to be good to fair, with nine studies rated as high quality^37–45^ (Table S2). All but one of the included studies stated the research questions or research objectives clearly.^46^ This study was, however, included as it provided information relevant to the review. Most studies lacked at least one item of the requisite information required by the MMAT. Among those studies providing all of the information required were one RCT,^36^ four observational mixed-methods studies,^38,42,44,47^ and four studies adopting qualitative methods.^39–41,45^

### Description of Studies

**Figure 1.**
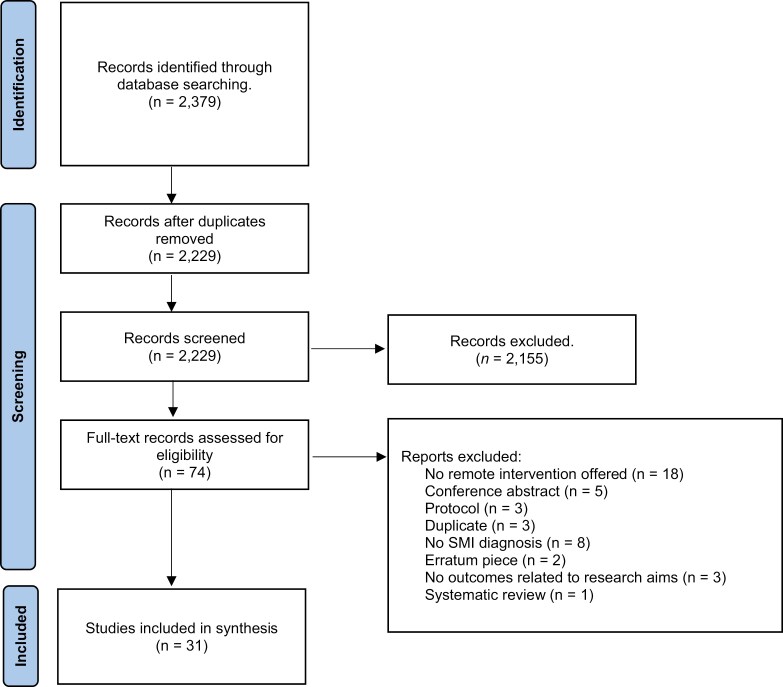
Preferred Reporting Items for Systematic Reviews and Meta-analyses diagram of paper selection process. SMI = severe mental illness.

The search identified 2379 articles; 31 studies were included in the review (Figure 1)^37–67^  Table S3 provides study characteristics, but in summary, 31 studies recruited a total of 3794 participants (range = 5–1787) using a variety of strategies. All but two^42,46^ of the studies were conducted in the United States. Fourteen studies adopted a randomized controlled design,^49–51,53,54,59–63,65–67^ of which four were definitive trials.^49,51,53,60^ Eight studies used mixed methods,^39,42–44,47,56–58^ five were qualitative studies,^38,40,41,45,46^ and three used a quantitative descriptive design.^48,52,55^ Lastly, one study adopted a nonrandomized controlled design.^64^ Almost all of the studies were set within community and outpatient mental health services, with just one offering an intervention to participants experiencing an inpatient admission.^49^

### Intervention Characteristics

Fifteen interventions designed to support adults with SMI were investigated in 24 studies. The remaining studies evaluated commercially available apps, online videos, and a variety of Websites. Among those interventions designed for the general population, three articles described interventions being tailored to the needs of people with SMI, with adaptations including providing information to address common beliefs and misapprehensions about smoking and mental illness,^48^ adapting motivational interviewing techniques to the needs of people with SMI,^49^ training of Quitline counselors to support callers with SMI,^49^ and ability to add information on mental health diagnosis into the app and receive a personalized tobacco dependence treatment plan which takes account of needs of people with SMI.^40^

The most frequent intervention described was the Learn To Quit intervention which was investigated in five studies.^44,47,50,59,66^ The Let’s Talk About Smoking intervention was explored in four studies,^37,51,54,67^ and the iCommit intervention in two.^45,63^ All but one of the interventions aimed to change the smoking behavior of people with SMI directly; however, the intervention developed by Aschbrenner et al.^48^ aimed to promote smoking cessation through the support of the family members of people with SMI. The majority of interventions were delivered via a mobile app,^38–41,43–45,47,50,58,59,62,66^ over the Internet,^37,51,52,54–56,60,61^ via telephone (including text messaging),^46,48,53^ or via video.^42^ Twenty-seven studies delivered interventions using one type of technology, while four^49,63–65^ studies delivered interventions using a combination of technology.

RCTs used varying controls, including usual care,^49,67^ apps or Web sites designed for the general public,^44,50,59–61,66^ informational resources delivered remotely,^51,55^ or enhanced usual care without the inclusion of a smartphone app.^63–65^ Further details relating to the interventions and control groups are presented in Table S4.

### What Is the Effectiveness of Digital Interventions in Supporting Smoking Cessation in Adults With SMI? (RQ1)

Four RCTs were excluded from the meta-analysis.^50,53,59,60^ Brunette et al.^53^ reported 7-day biochemically verified smoking abstinence but did not report the results separately for the control group versus the two intervention arms; Heffner et al.^60^ reported 30-day point prevalence abstinence for the whole sample and only separated results by diagnosis; Halverson et al.^59^ did not report smoking cessation-related outcomes, and Browne et al.^50^ reported a reduction in cigarette consumption rather than smoking abstinence.

Due to the nature of digitally delivered interventions, where there is often no face-to-face contact and biochemical confirmation may not be feasible, RCTs using self-reported cessation outcomes were also included in the meta-analysis. Subgroup analyses to separate those that included biochemically verified smoking abstinence, and those that included self-reported smoking abstinence were not considered due to the number imbalance (8 of 9 studies included in the meta-analysis included biochemically verified smoking abstinence).

**Figure 2.**
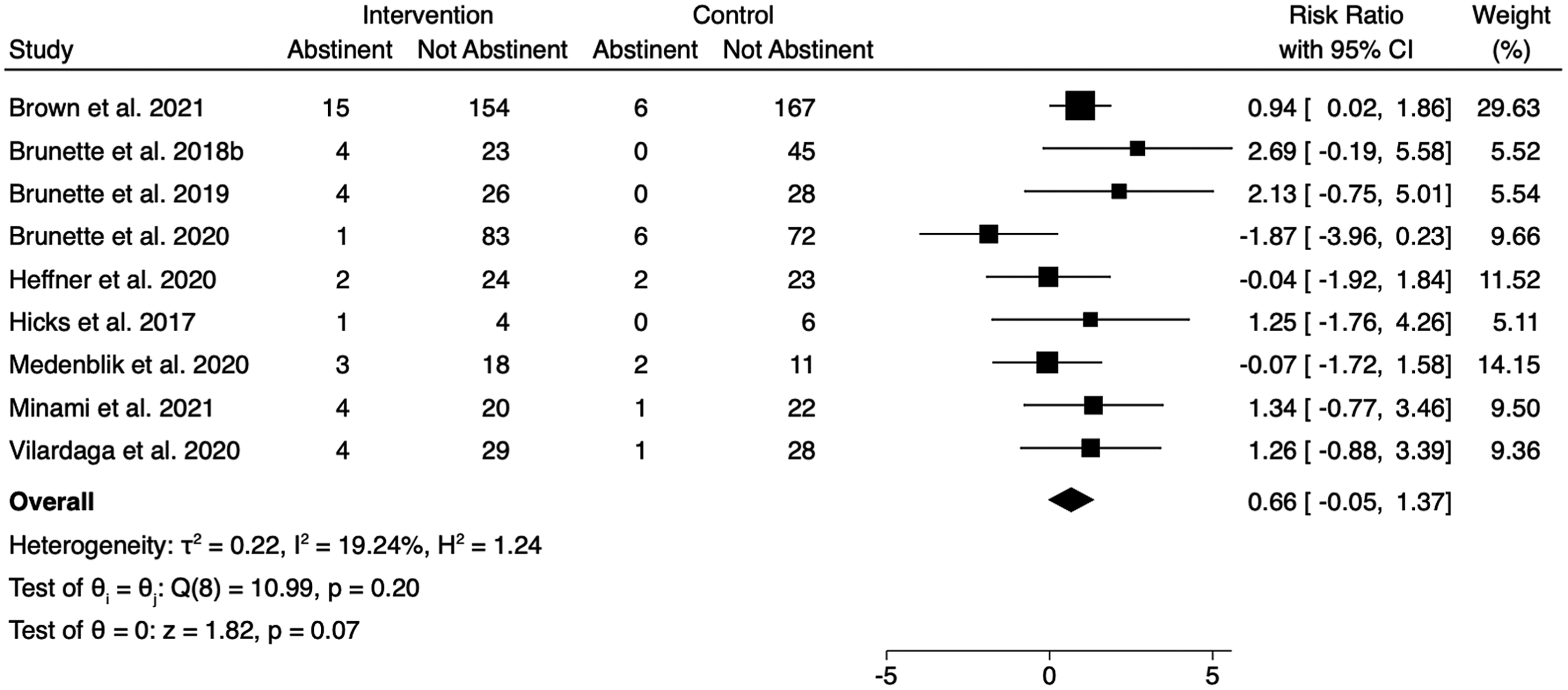
Comparison of abstinence (biochemically verified or self-reported) at longest follow-up in randomised controlled trials . CI = confidence interval. RR are presented on the log scale.

The meta-analysis of smoking abstinence at longest respective follow-up (month 1 to month 6) did not find an overall effect in favor of intervention (log risk ratio [Log RR] = 0.66, 95% CI = −0.005 to 1.37; Figure 2), corresponding to a RR of 1.94 (95% CI = 1.0 to 3.94). Overall, 9.1% of participants in the intervention groups (38/419) achieved abstinence compared with 4.3% in the control groups (18/420).

**Figure 3.**
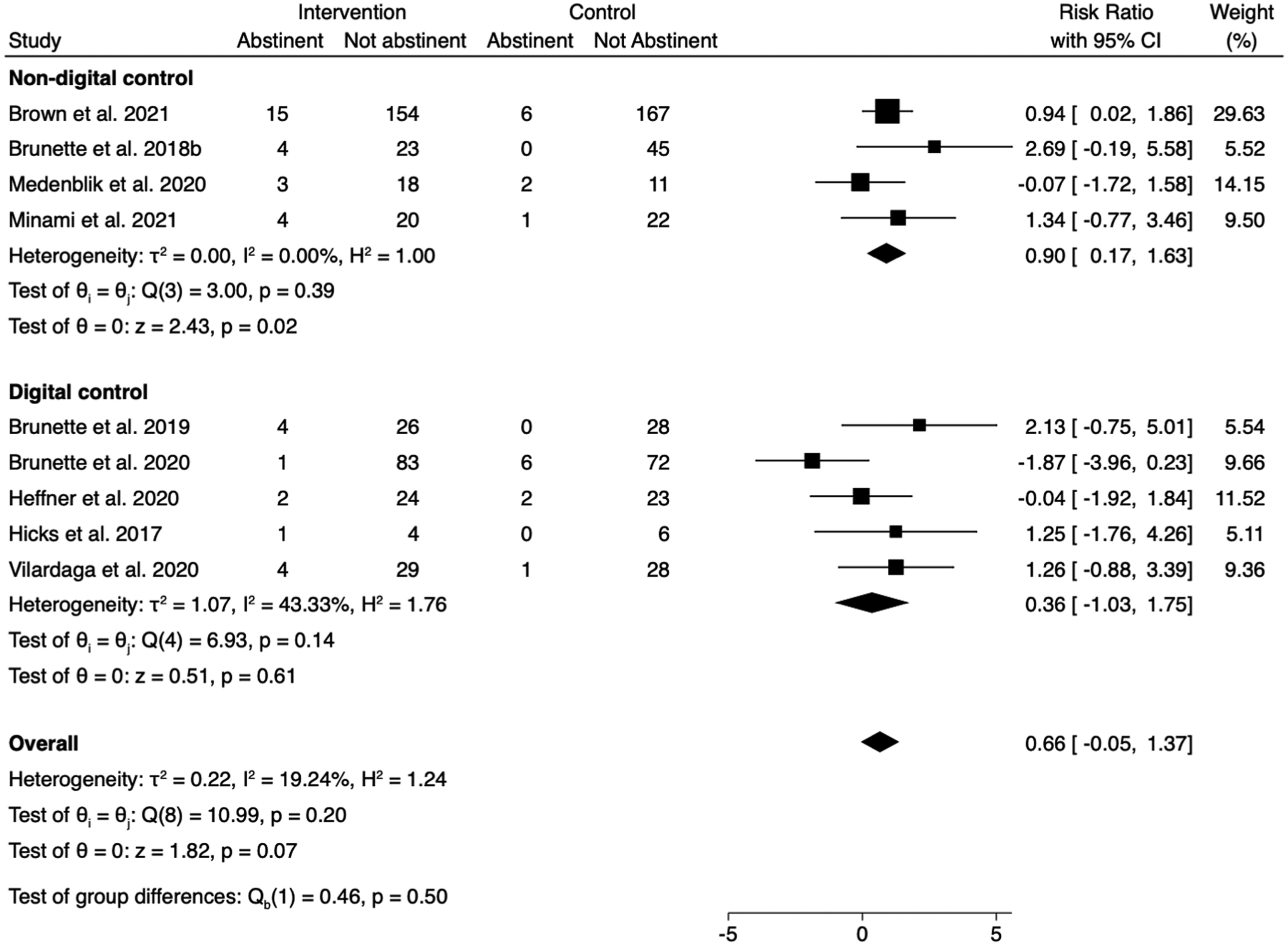
Comparison of abstinence (biochemically verified or self-reported) in randomised controlled trials with a nondigital control group versus a digital control group. CI = confidence interval. RR are presented on the log scale.

Subgroup analyses were conducted to separate studies by those that included nondigital control groups from those that included digital control groups (Figure 3). The subgroup analysis of smoking abstinence in studies with a nondigital control group did not find an overall effect in favour of intervention (log RR = 0.90, 95% CI = 0.17 to 1.63), corresponding to an RR of 2.46 (95% CI = 1.19 to 5.10). Likewise, the subgroup analysis of smoking abstinence in studies with a digital control group did not find an overall effect in favor of intervention (log RR = 0.36, 95% CI = −1.03 to 1.75), corresponding to an RR of 1.43 (95% CI = 0.36 to 5.75). There was no difference in the log RRs for those with a digital or nondigital control group, based on the overlapping CIs.

Asymmetry in the funnel plot indicates the possibility of publication bias (Figure S1); however, Egger’s regression intercept was not significant (*p* > .05), so the trim-and-fill method was not used.

### What are Participant, Caregiver, and/or Staff Experiences of Digital Interventions to Support Smoking Cessation in Adults With SMI? (RQ2)

#### Acceptability

Thirteen studies reported qualitative data describing participant experiences of receiving digital interventions. While participants were overall positive about the interventions, a range of barriers to engagement were reported, primarily linked to reduced levels of digital familiarity and literacy among participants.^41,57,58^ Participants perceived mobile interventions to be a convenient, instantly accessible, helpful, and an easy-to-use technology, with the advantage of anonymity.^39,41,44,47,55^ Smartphone apps and videos were also perceived as positive and nonjudgmental by participants, with some commenting on the supportive, almost caring tone adopted by the intervention.^39,40,42^ One study noted the importance of a caring tone and expressed concern that a negative tone could undermine their quit attempts by evoking feelings of guilt and failure.^39^ Additionally, a study found that when working with family members of people with SMI who smoke, it is important to be aware of the broader social and illness-related context surrounding the individual with SMI and to be flexible in the approach to intervention.^48^

The importance of tailoring an intervention to participants needs was highlighted.^40,42^ Participants considered personal autonomy important and appreciated interventions that did not prescribe a “one-size-fits-all” approach.^42^ When asked to make recommendations for improvements to interventions, many participants expressed a desire for greater personalization.^39,41,43,58^ For example, in one study,^39^ participants stated that they preferred keeping track of their daily cigarette counts to monitor their progress in quitting smoking. They believed this approach was more positive and helpful than tracking lapses to smoking or smoke-free days since it allowed them to frame their personal progress positively.

Among studies exploring the content and functionality of digital interventions, a variety of perceptions were obtained from participants. Gamification and achieving a sense of “winning” were important to participants.^39,43,44,47^ Furthermore, the inclusion of quizzes and learning within apps was suggested to increase motivation^43^ assist participants in retaining and recalling the content of apps,^44^ and serve as a distraction from cravings.^38^ For example, one participant stated, “that would be good, like a medal […], like in a game, you have first place on this level.”^43^ Interventions in two studies contained “chatroom” features which provided contrasting acceptability findings. In the first study, participants expressed little interest in connecting with others on a social platform about their quit attempts, believing it to be detrimental if they were not successful.^39^ However, some believed that such a platform would be beneficial and facilitate connections with others, from whom they could derive support and a shared understanding of the challenges involved in quitting.^39^ In the second study, participants expressed enthusiasm for engagement through the app, particularly the idea of a chatroom specific to mental illness and smoking where they could connect with like-minded people.^40^ Finally, the graphics used within interventions provoked mixed perceptions by participants. For the majority, simple cartoon-based graphics and visual storytelling were considered “eye-catching” and were well-received.^44,47^ However, for the minority, it caused anxiety and irritation.^42^ Videos using a documentary format achieved positive responses, in particular if they included real life, as opposed to cartoon presenters, and the inclusion of expert onion.^42^

#### Usability

Five studies investigated usability through the administration of the System Usability Scale (SUS).^39,43,44,47,66^ Variability in SUS scores was observed, with three studies recording usability above the 68-point cutoff,^44,47,66^ and two studies fell below the cutoff.^39,43^ Authors commented, however, that while the SUS cutoff was not met, three-quarters of the participants reported the intervention as easy to use.^39^

The Perceived Usefulness and Ease of Use scale was utilized in five studies.^38,51,52,55,57^ Overall, scores indicated that with the tailoring of the intervention, participants were able to easily understand the information presented^51,57,58^ and gain proficiency over the course of the intervention,^57^ thereby increasing perceptions of usefulness.^58^

In their examination of neurocognition and social cognition, Halverson and colleagues found users of the Learn To Quit app had significantly more interactions than the control group.^59^ However, patient characteristics, including those related to clinical diagnosis, as well as neurocognition and social cognition were not found to be associated with app engagement. Both experiential avoidance and symptom severity were found to influence the time spent interacting with the app.^59^ Higher scores on the Avoidance and Inflexibility Scale were identified as being a significant predictor of the duration of app engagement, while higher scores on the Brief Symptom Inventory predicted a shorter duration of engagement.^59^

Barriers to using digital interventions were identified by Brunette et al.^52,55^ In the first instance, minimal prior experience in using a computer meant that none of the four Web sites being tested could be navigated by participants, and just one Web site could be navigated by participants with limited experience.^52^ Secondly, when accessing an app-based intervention, two (10%) participants were observed to experience challenges in the use of a mobile smoking cessation intervention directly apportioned to their SMI diagnosis.^55^ For one participant, difficulties with information processing and manual dexterity could not be overcome with additional coaching causing the participant to complete the intervention with the assistance of their therapist. For another participant, paranoia related to the use of the computer prevented completion of the intervention. However, videos promoting smoking cessation were found to increase knowledge and confidence in quitting by approximately 80%.^42^

### What Are the Factors Influencing Implementation and Delivery of Digital Interventions for Adults With SMI? (RQ3)

Of the 31 included articles, 10 provided information on implementing and delivering digital interventions. These strategies sought to mitigate any logistical challenges or burdens that participants may experience. Studies offered training in the form of practice sessions to familiarize participants with devices and software,^39,43,56^ offered the opportunity for participants to attend a weekly technical coaching session to troubleshoot technical problems and improve their ability to use smartphone technology,^44^ included a coaching feature within the app-based intervention,^47^ or offered technical support to participants informed by the supportive accountability model, which has been shown to be particularly beneficial in SMI populations.^66^

Where family members were trained to promote the use of a digital cessation decision aid for smokers with SMI, relatives were provided with a coaching session to understand the purpose of the intervention and to build confidence in using the motivational interviewing approach.^48^ Furthermore, the authors identified that partnering with third-sector and patient advocacy groups encouraged trust in the relatives and use by the smokers themselves.^48^ Implementation was also supported through the loaning of devices and monetary incentivization for the collection of outcome data. Five studies reported using monetary reinforcement ranging between $205 and $530 paid to participants submitting carbon monoxide measurements throughout the intervention period.^43,56,62–64^ Five studies provided participants with devices inclusive of airtime and data to facilitate participants’ engagement with the intervention.^43,45,47,64,66^ However, one study by Vilardaga et al.^66^ found that many participants owned mobile phones and were familiar with smartphone technology. Furthermore, the authors chose to deliver the intervention via an Android platform, as it has been identified in formative work to be the preferred operating system for individuals with SMI.^47^

### How Well-Reported Are Digital Interventions to Support Smoking Cessation in Adults With SMI? (RQ4)

Twenty-five articles were evaluated for quality and completeness of the reporting of interventions. Six studies were excluded from the analysis as they evaluated existing smoking cessation interventions.^38,43,44,47,58,59^ Overall, interrater reliability was calculated as a statistically significant strong level of agreement (*k* 0.945 [95% CI = 0.880 to 0.970], *p* < .001).

Assessment using the TIDiER checklist^35^ found considerable variability in intervention reporting, with six (24.0%) of the 25 included studies reporting all of the information expected. Items most likely to be responded to included intervention name (100%), intervention rationale (100%), and mode of intervention delivery (96%). Those least likely to be responded to include items relating to the provider of the intervention (54%), the nature of any tailoring or in-trial modification of the intervention (37%), and assessments of intervention acceptability or fidelity (25%).

## Discussion

To our knowledge, this is the first evidence synthesis study to estimate the overall effectiveness of digital smoking cessation interventions for adults with SMI. It is also the first study to investigate the experience of implementing and receiving such interventions, and those factors which influence successful implementation.

Thirty-one studies were identified, describing the development, testing, and delivery of 24 distinct interventions. Aligning with findings in the general population^20^ and meta-analyses of interventions targeting adults with SMI who smoke,^68^ our meta-analysis did not find a statistically significant effect for digital smoking cessation interventions delivered to people with SMI. However, due to the limited number of RCTs available, relatively small sample sizes, heterogeneity of study quality, and variation in the types of digital interventions provided, it is challenging to interpret the current meta-analysis results in terms of their promise for individuals with SMI. Despite this, the current review did indicate that overall, 9.1% (38/419) of participants in the intervention groups achieved abstinence compared with 4.3% (18/420) in the control groups, pointing toward opportunities to improve future research in this area. This aligns with a recent narrative synthesis conducted by Agullerio et al.^26^ that indicated digital smoking cessation interventions may be a suitable and promising alternative for people with SMI, reporting abstinence rates ranging between 9% and 40%. However, the focus on determining effectiveness raises an important issue: reporting of digital smoking cessation interventions is often insufficiently accurate, comprehensive, and transparent. For example, authors often did not report data on the intervention provider, the nature of any tailoring, and assessments of intervention acceptability or fidelity. Inadequate reporting can make it challenging for researchers to replicate trials, for intervention developers to design effective interventions, and for providers to implement interventions in practice.^69^ No existing systematic reviews have formally evaluated the quality and completeness of reporting these digital smoking cessation interventions for this population by assessing reporting in accordance with gold standard reporting guidelines. Therefore, the current results address this gap in the literature and further emphasize the need for more rigorous RCTs in the area. Our findings also contrast with reported effectiveness for established and evidence-based smoking cessation support (behavioral support plus pharmacotherapy), as previous reviews have concluded pharmacotherapy for smoking cessation in people with SMI is safe, acceptable, and effective.^68,70,71^ However, trials exploring the effectiveness of digital interventions differ from pharmacological trials in that they examine the effectiveness of a digital intervention (eg, an app) compared to no or an alternative digital intervention, regardless of access to pharmacotherapy. Therefore, it is difficult to recommend whether smoking cessation interventions for people with SMI should include a digital element alongside pharmacotherapy.

While we found no evidence for digital technology elements being effective in supporting smoking cessation (alongside pharmacotherapy), quantitative and qualitative findings suggest that digitally delivered interventions are an acceptable form of smoking cessation support for this population. This builds on findings reported by Agullerio et al.,^26^ as the authors highlighted high acceptability rates for a range of digital interventions. However, these findings were primarily based on quantitative scores from author-developed scales and were limited by the data available in the included studies. Our current findings extend previous insights into the perceptions of digital smoking cessation interventions, markedly, highlighting the importance of flexibility and tailoring the intervention to individual needs within this population group. For example, Halverson et al. reported that assignment to the tailored intervention group was the strongest predictor of app engagement after controlling for demographic, clinical, and cognitive characteristics.^59^ These results also draw attention to the importance of a user-centered design approach to intervention development, with studies reporting increased usability scores for interventions applying these principles.^40,43–45,47,50,55–57^ This is in line with previous works which found implementation of a user-centered approach to design was a strong predictor of engagement, and further underscores the need to utilize such an approach when developing or tailoring interventions to support smoking cessation to people with SMI.^72,73^

This finding aligns with the recent exploration of smoking cessation apps tailored to adults with mental health conditions who smoke by Chen et al.,^74^ who reported that while being unable to conclusively determine evidence of effect, mainly due to methodological heterogeneity, the authors reported that apps grounded in research are generally perceived as effective by their users. In addition to methodological heterogeneity, potential observed differences may also be related to heterogeneity relating to participant characteristics, including diagnosis, severity of mental illness, and the type of treatment being received, as well as the presence of comorbidity.^75^ Furthermore, in common with other authors, limited reporting of relevant information on the characteristics of participants impeded our assessment of clinical heterogeneity.^76^

Synthesis of quantitative and qualitative data demonstrated that people with SMI have an interest in using digitally delivered smoking cessation interventions despite concerns that challenges relating to digital literacy and cognition may negatively impact interest and engagement.^77,78^ Our results suggest that users with a range of SMI and cognitive abilities are motivated and able to engage with interventions on digital platforms. While we found little indication that a diagnosis of SMI was a barrier, samples were drawn from a community population, and the impact of acuity on participant engagement is unknown. While the findings suggest that the provision of devices and monetary reinforcement was an enabler to the research, this may present a challenge when interventions are scaled, both in terms of real-world implementation and in making comparisons of effectiveness. However, despite many studies finding insignificant differences in the long term, monetary reinforcement is considered a promising short-term approach in supporting abstinence in people with schizophrenia who smoke.^79^

While it appeared that all of the interventions were successfully implemented, reporting in relation to this was limited and largely focused on overcoming practical barriers at the participant level. One study mentioned implementation being underpinned by theory; however, this only related to the provision of technical support.^66^ Implementation interventions are complex strategies designed to improve clinical interventions and change behaviors at the organizational, practitioner, or patient levels.^80^ To ensure that important factors are not overlooked in the implementation process and to understand the reasons behind the success or failure of an intervention, future research should develop theory-informed strategies to guide implementation.^81^ This linkage between theory and outcomes is crucial for effective implementation.

With digital health technologies increasingly being deployed as an adjunct to health interventions, further research is needed to identify and understand the factors that facilitate or hinder the implementation of digital smoking cessation interventions for people with SMI. Prioritizing barriers and linking strategies to overcome them is essential. The use of implementation theories and frameworks such as Normalization Process Theory or the Consolidated Framework for Implementation Research, as well as the Behavior Change Wheel, can assist in implementation planning and evaluation, as well as the identification of the dynamics of embedding interventions at multiple levels.^82–84^

### Limitations and Strengths

Variations in methodology and reporting mean that the findings from this review should be interpreted cautiously. While a comprehensive search of bibliographic databases was undertaken, it is possible that some studies were missed. It is debatable whether this would have affected the overall conclusion of this review. We found the quality of studies overall to be good to fair. This contrasts with an earlier review^20^ of digital smoking cessation interventions within the general population, which were found to be below average and prompted calls for increased transparency and uniformity in the conduct and reporting of such trials. There is also some evidence of potential publication bias in this review, as indicated by the forest plot asymmetry, which is likely due to the inclusion of several studies with small sample sizes. Within this review, subgroup analyses were undertaken to assess the effect of studies comparing the intervention with digital and nondigital controls, which included active controls. However, owing to limited descriptions, the quality of comparison conditions could not be established. In relation to describing the tailoring of interventions, over two-thirds of the studies included in this review failed to provide justification and descriptions of the process for tailoring interventions to people with SMI population.

The studies reviewed suggest that there has been a gradual increase in the use of smoking cessation interventions for individuals with SMI over time. This growth has been driven by advancements in technology. Although it is still unclear whether digital interventions are effective in helping adults with SMI quit smoking, ongoing and future research will likely lead to a better understanding of their effectiveness.

Given the emergent evidence on the effectiveness of tailored interventions, further research, particularly RCTs with larger sample sizes, is required. Additionally, in the absence of guidance on the reporting of studies evaluating digital smoking cessation interventions, authors should strive for the rigorous reporting of the tailored aspects of the intervention as well as controls.

## Conclusions

This review highlights the growing evidence underpinning digital interventions to support smoking cessation among people with SMI and finds that such interventions when tailored to people with SMI appear promising in terms of effectiveness and acceptability. While some facilitators to implementation were discussed by authors, the impact of strategies deployed is unknown. We, therefore, recommend that future research explore the receipt of implementation strategies as well as the intervention. Furthermore, rigorous reporting of the tailored aspect of interventions is recommended.

## Supplementary Material

Supplementary material is available at *Nicotine and Tobacco Research* online.

ntae237_suppl_Supplementary_Figure_S1

ntae237_suppl_Supplementary_Table_S1

ntae237_suppl_Supplementary_Table_S2

ntae237_suppl_Supplementary_Table_S3

ntae237_suppl_Supplementary_Table_S4

## Data Availability

The data sets used in this study can be found in the full-text articles included in the systematic review and meta-analysis.
