# Correction to: Effectiveness and Experience of Implementing Digital Interventions to Promote Smoking Cessation Among Adults With Severe Mental Illness: A Systematic Review and Meta-analysis

**DOI:** 10.1093/ntr/ntag049

**Published:** 2026-06-18

This is a correction to: Lisa Huddlestone, Emily Shoesmith, Jodi Pervin, Rosie Stevens, Simon Gilbody, Elena Ratschen, Effectiveness and Experience of Implementing Digital Interventions to Promote Smoking Cessation Among Adults With Severe Mental Illness: A Systematic Review and Meta-analysis, *Nicotine & Tobacco Research*, Volume 27, Issue 6, June 2025, Pages 951–961, https://doi.org/10.1093/ntr/ntae237

Following publication, the authors identified a reporting issue in several sections of the article. Specifically, the meta-analyses were conducted using log-transformed risk ratios (Log RRs), which is methodologically appropriate. However, this was not clearly stated in the original text or figure captions. In some instances, the effect estimates were mistakenly reported as raw risk ratios (RRs).

To resolve this, the following corrections have been made to the article, to indicate that results are based on log-transformed RR and to provide exponentiated values (RR) alongside the log RRs.

In the Results section of the Abstract, in the following sentence, “(risk ratio = 0.66, 95% confidence interval = −0.005 to 1.37)” has been amended to read “(log risk ratio = 0.66, 95% confidence interval = −0.005 to 1.37)”:

“Meta-analysis of biochemically verified abstinence at longest follow-up (month 1 to month 6) did not find an overall effect in favour of intervention (risk ratio = 0.66, 95% confidence interval = −0.005 to 1.37).”

The amended sentence reads:

“Meta-analysis of biochemically verified abstinence at longest follow-up (month 1 to month 6) did not find an overall effect in favour of intervention (log risk ratio = 0.66, 95% confidence interval = −0.005 to 1.37).”

In the RQ1 Results section, the following text has been corrected:

“The meta-analysis of smoking abstinence at longest respective follow-up (month 1 to month 6) did not find an overall effect in favor of intervention (risk ratio [RR] = 0.66, 95% CI = −0.005 to 1.37; Figure 2). Overall, 9.1% of participants in the intervention groups (38/419) achieved abstinence compared with 4.3% in the control groups (18/420).

Subgroup analyses were conducted to separate studies by those that included nondigital control groups from those that included digital control groups (Figure 3). The subgroup analysis of smoking abstinence in studies with a nondigital control group did not find an overall effect in favor of intervention (RR = 0.90, 95% CI = 0.17 to 1.63). Likewise, the subgroup analysis of smoking abstinence in studies with a digital control group did not find an overall effect in favor of intervention (RR = 0.36, 95% CI = −1.03 to 1.75). There was no difference in the RRs for those with a digital or nondigital control group, based on the overlapping CIs.”

The corrected text reads:

“The meta-analysis of smoking abstinence at longest respective follow-up (month 1 to month 6) did not find an overall effect in favor of intervention (log risk ratio [Log RR] = 0.66, 95% CI = −0.005 to 1.37; Figure 2), corresponding to a RR of 1.94 (95% CI = 1.0 to 3.94). Overall, 9.1% of participants in the intervention groups (38/419) achieved abstinence compared with 4.3% in the control groups (18/420).

Subgroup analyses were conducted to separate studies by those that included nondigital control groups from those that included digital control groups (Figure 3). The subgroup analysis of smoking abstinence in studies with a nondigital control group did not find an overall effect in favour of intervention (log RR = 0.90, 95% CI = 0.17 to 1.63), corresponding to an RR of 2.46 (95% CI = 1.19 to 5.10). Likewise, the subgroup analysis of smoking abstinence in studies with a digital control group did not find an overall effect in favor of intervention (log RR = 0.36, 95% CI = −1.03 to 1.75), corresponding to an RR of 1.43 (95% CI = 0.36 to 5.75). There was no difference in the log RRs for those with a digital or nondigital control group, based on the overlapping CIs.”

Finally, the captions of Figure 2 and 3 have been corrected to add the sentence: “RR are presented on the log scale.”

