# Supplementary figures and images for: Effectiveness and Experience of Implementing Digital Interventions to Promote Smoking Cessation Among Adults With Severe Mental Illness: A Systematic Review and Meta-analysis
Source: Nicotine Tob Res. 2024 Oct 9;27(6):951–61. doi: 10.1093/ntr/ntae237 (PMC12095810; doi:10.1093/ntr/ntae237)

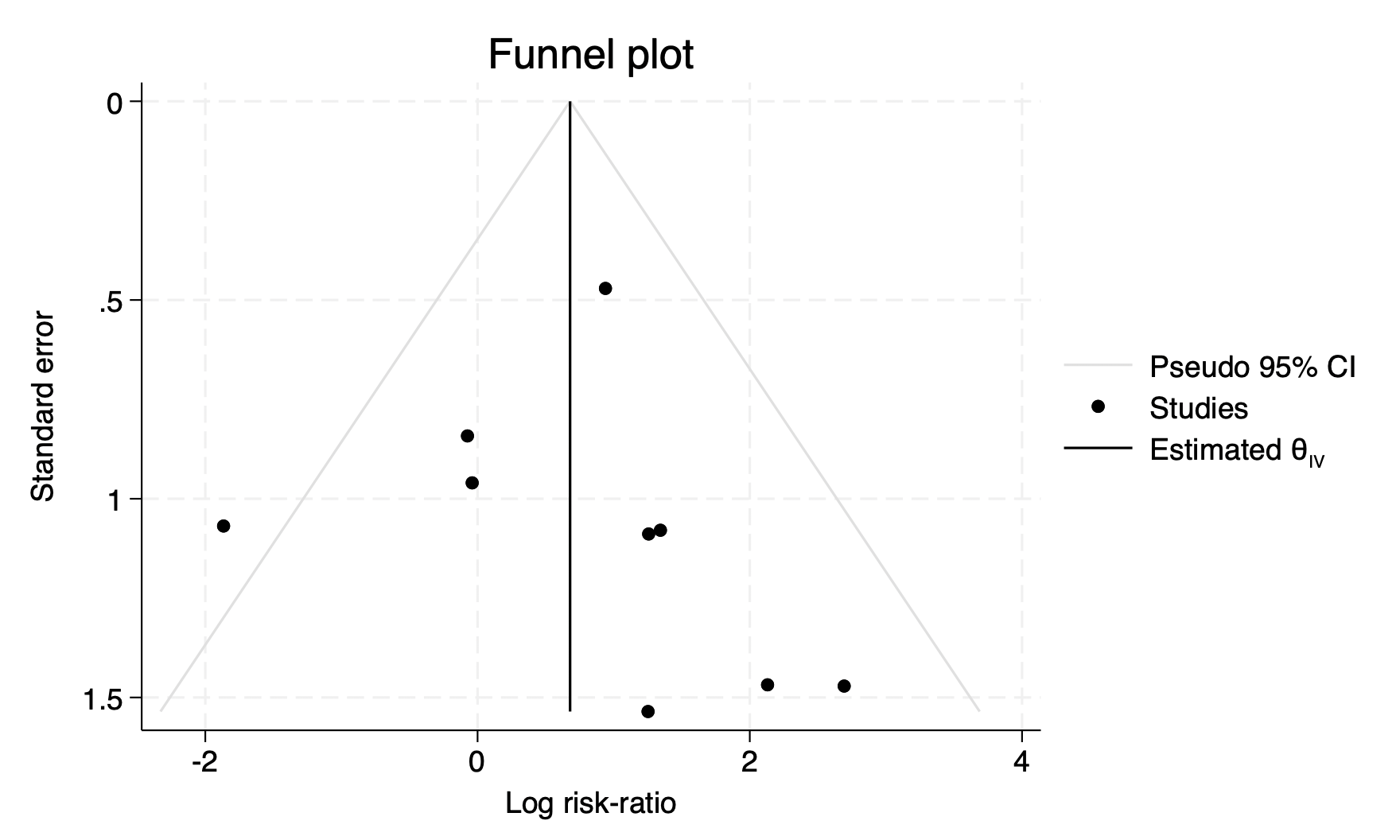
**Supplementary Figure 1.** Funnel plot of studies included in the meta-analysis

Supplement: ntae237_suppl_Supplementary_Figure_S1 [file ntae237_suppl_supplementary_figure_s1.docx]
