# Supplementary material for: Effectiveness and Experience of Implementing Digital Interventions to Promote Smoking Cessation Among Adults With Severe Mental Illness: A Systematic Review and Meta-analysis
Source: Nicotine Tob Res. 2024 Oct 9;27(6):951–61. doi: 10.1093/ntr/ntae237 (PMC12095810; doi:10.1093/ntr/ntae237)
Supplement: ntae237_suppl_Supplementary_Table_S1 [file ntae237_suppl_supplementary_table_s1.docx]

**Supplementary Table 1. Search strategy and search terms**

| **Search terms** | |
| --- | --- |
| **Population** | (Severe mental illness OR serious mental illness OR SMI OR Schizophrenia OR bipolar disorder OR major depressive disorder OR major depression OR depression OR schizoaffective disorder OR psychosis or post-traumatic stress disorder OR PTSD) |
| **AND** | |
| **Intervention** | Digital OR web OR app OR application or smartphone OR mhealth OR mobile health OR ehealth OR mobile device OR internet |
| **AND** | |
| **Outcome** | (Smoking OR tobacco) AND (cessation OR quit* OR abstinence OR harm reduction) OR Perspect* OR belief* OR attitude* OR view* OR experien* OR implement* OR acceptability OR usability OR |
