# Supplementary material for: Effectiveness and Experience of Implementing Digital Interventions to Promote Smoking Cessation Among Adults With Severe Mental Illness: A Systematic Review and Meta-analysis
Source: Nicotine Tob Res. 2024 Oct 9;27(6):951–61. doi: 10.1093/ntr/ntae237 (PMC12095810; doi:10.1093/ntr/ntae237)
Supplement: ntae237_suppl_Supplementary_Table_S2 [file ntae237_suppl_supplementary_table_s2.docx]

**Supplementary Table 2. Results of MMAT appraisal**

| **QUALITATIVE** | | | | | | | |
| --- | --- | --- | --- | --- | --- | --- | --- |
| **Author (date)** | **Screening** | | **MMAT criteria** | | | | |
|  | **Are there clear research questions?** | **Do the collected data allow to address the research questions?** | **Is the qualitative approach appropriate to answer the research question?** | **Are the qualitative data collection methods adequate to address the research question?** | **Are the findings adequately derived from the data?** | **Is the interpretation of results sufficiently substantiated by data?** | **Is there coherence between qualitative data sources, collection, analysis, and interpretation?** |
| Gowarty (2020) | Yes | Yes | Yes | Yes | Yes | Yes | Yes |
| Herbst (2019) | Yes | Yes | Yes | Yes | Yes | Yes | Yes |
| Klein (2019) | Yes | Yes | Yes | Yes | Yes | Yes | Yes |
| Leutwyler (2021) | No | Can’t tell | Can’t tell | Can’t tell | Yes | Yes | Yes |
| **QUANTITATIVE RANDOMISED CONTROLLED TRIALS** | | | | | | | |
| **Author (date)** | **Screening** | | **MMAT criteria** | | | | |
|  | **Are there clear research questions?** | **Do the collected data allow to address the research questions?** | **Is randomization appropriately performed?** | **Are the groups comparable at baseline?** | **Are there complete outcome data?** | **Are outcome assessors blinded to the intervention provided?** | **Did the participants adhere to the assigned intervention?** |
| Brown (2021) | Yes | Yes | Yes | Yes | Yes | Can’t tell | Can’t tell |
| Browne (2021) | Yes | Yes | Yes | Yes | No | Can’t tell | Can’t tell |
| Brunette (2020) | Yes | Yes | Yes | Yes | No | Yes | Yes |
| Brunette (2011) | Yes | Yes | Yes | Yes | Yes | Yes | Yes |
| Brunette (2018) | Yes | Yes | Yes | Yes | No | Can’t tell | Yes |
| Brunette (2019) | Yes | Yes | Yes | Yes | No | Can’t tell | Can’t tell |
| Halverson (2022) | Yes | Yes | Can’t tell | Yes | Yes | Can’t tell | Can’t tell |
| Heffner (2018) | Yes | Yes | Can’t tell | Yes | Yes | Can’t tell | Can’t tell |
| Heffner (2020) | Yes | Yes | Can’t tell | Yes | Yes | Yes | Yes |
| Hicks (2017) | Yes | Yes | Can’t tell | Yes | No | Can’t tell | Yes |
| Medenblik (2020) | Yes | Yes | Yes | Yes | No | Yes | Yes |
| Minami (2021) | Yes | Yes | Yes | Yes | Yes | Can’t tell | Yes |
| Vilardaga (2020) | Yes | Yes | Can’t tell | No | Yes | Can’t tell | Yes |
| **QUANTITATIVE NON-RANDOMISED** | | | | | | | |
| **Author (date)** | **Screening** | | **MMAT criteria** | | | | |
|  | **Are there clear research questions?** | **Are there clear research questions?** | **Are the participants representative of the target population?** | **Are measurements appropriate regarding both the outcome and intervention (or exposure)?** | **Are there complete outcome data?** | **Are the confounders accounted for in the design and analysis?** | **Is the intervention administered (or exposure occurred) as intended?** |
| Aschbrenner (2018) | Yes | Yes | Yes | Yes | Can’t tell | Can’t tell | Yes |

| **QUANTITATIVE DESCRIPTIVE** | | | | | | | |
| --- | --- | --- | --- | --- | --- | --- | --- |
| **Author (date)** | **Screening** | | **MMAT criteria** | | | | |
|  | **Are there clear research questions?** | **Are there clear research questions?** | **Is the sampling strategy relevant to address the research question?** | **Is the sample representative of the target population?** | **Are the measurements appropriate?** | **Is the risk of nonresponse bias low?** | **Is the statistical analysis appropriate to answer the research question?** |
| Brunette (2012) | Yes | Yes | Yes | No | Yes | Yes | Can’t tell |
| Brunette (2019) | Yes | Yes | Yes | No | Yes | Yes | Can’t tell |
| Minami (2018) | Yes | Yes | Yes | No | Yes | Yes | Can’t tell |
| Wilson (2019) | Yes | Yes | Yes | Yes | Yes | Yes | Yes |
| **MIXED METHODS** | | | | | | | |
| **Author (date**) | **Screening** | | **MMAT criteria** | | | | |
|  | **Are there clear research questions?** | **Are there clear research questions?** | **Is there an adequate rationale for using a mixed methods design to address the research question?** | **Are the different components of the study effectively integrated to answer the research question?** | **Are the outputs of the integration of qualitative and quantitative components adequately interpreted?** | **Are divergences and inconsistencies between quantitative and qualitative results adequately addressed?** | **Do the different components of the study adhere to the quality criteria of each tradition of the methods involved?** |
| Brunette (2016) | Yes | Yes | Yes | Yes | Can’t tell | Can’t tell | Can’t tell |
| Ferron (2017) | Yes | Yes | Yes | No | No | No | No |
| Ferron (2011) | Yes | Yes | Yes | Yes | Yes | Can’t tell | Can’t tell |
| Gowarty (2021) | Yes | Yes | Yes | Yes | Yes | Yes | Yes |
| Sharma-Kumar (2021) | Yes | Yes | Yes | Yes | Yes | Yes | Yes |
| Vilardaga (2016) | Yes | Yes | Yes | Yes | Yes | Yes | Yes |
| Vilardaga (2019) | Yes | Yes | Yes | Yes | Yes | No | Yes |
| Vilardaga (2018) | Yes | Yes | Yes | Yes | Yes | Yes | Yes |
