# Supplementary material for: Effectiveness and Experience of Implementing Digital Interventions to Promote Smoking Cessation Among Adults With Severe Mental Illness: A Systematic Review and Meta-analysis
Source: Nicotine Tob Res. 2024 Oct 9;27(6):951–61. doi: 10.1093/ntr/ntae237 (PMC12095810; doi:10.1093/ntr/ntae237)
Supplement: ntae237_suppl_Supplementary_Table_S3 [file ntae237_suppl_supplementary_table_s3.docx]

**Supplementary table 3. Study characteristics**

| **STUDY** | | | | **PARTICIPANTS** | | | | | | **QUALITY ASSESSMENT** |
| --- | --- | --- | --- | --- | --- | --- | --- | --- | --- | --- |
| **Author (Year)**  **Country** | **Study design** | **Primary outcome** | **Secondary outcome** | **Diagnosis** | **Sample size**  **(intervention/control)** | **Sex (%), age mean (SD)** | **Setting** | **FTND score mean (SD)** | **Cigarettes per day mean (SD)** | **MMAT Appraisal** |
| Aschbrenner (2018)  United States | Non-randomised feasibility study | Smoker's use of online decision-aid | - | SZ, BPD, MDD, PTSD, AD | 34 | Male (88%)  38 years (13.5) | Community | - | - | Good |
| Brown  (2021)  United States | RCT | Self-reported abstinence: 7-day PPA verified at 6 months | - | MDD, BPD, PTSD, SZ, PD, ED, AD, OCD, CD, ND, NC, SD | 353  (169/173) | Male 53%  36 years (12.4) | Inpatient | 4.7 | 17.0 | Fair |
| Browne (2021)  United States | Pilot RCT | App interactions | - | MDD, BPD, SZ | 62 (33/29) | Female 64%  46 years (11.3) | Community | - | 21.0 | Good |
| Brunette  (2011)  United States | Quasi-experimental | Use of smoking cessation treatment | - | SZ | 42  (21/20) | - | Outpatient | - | - | High |
| Brunette (2012)  United States | Quantitative | Usability | - | MDD, BPD, SZ | 16 | Male (62%)  49 years (8.9) | Community | - | - | Good |
| Brunette  (2016)  United States | Quantitative evaluation | Usability | - | SZ | 11 | Female (55%)  49 years (12.0) | Community | - | 13.0 | Fair |
| Brunette  (2018)  United States | RCT | Use of smoking cessation treatment  Quit attempts | Abstinence  User satisfaction | SZ, MD, AD | 81  (30/28/23) | Male (63%)  24 years (3.6) | Community | 4.6 (2.0) | 14.2 (11.6) | Good |
| Brunette  (2018)  United States | RCT | Self-reported abstinence: 7-day PPA | Self-reported tobacco consumption at 3-months | SZ, MDD, BPD, AD | 661  (PV+CBT = 212 / PV+Q = 303 / Control = 146) | Female (64%)  45 years (10.8) | Community | 5.3 (2.3) | 17.3 (10.5) | Good |
| Brunette  (2019)  United States | Pre/post study | Feasibility  Efficacy | - | SZ, MDD, BPD | 20 | - | Outpatient | - | - | Good |
| Brunette (2019)  United States | RCT - Secondary analysis | Abstinence | Treatment attitudes  Use of smoking cessation treatment,  Quit attempts | SZ, MD, AD | 58  (30/28) | Male (63%)  24 years (3.6) | Community | 4.6 (2.0) | - | Fair |
| Brunette; (2020)  United States | RCT | Use of smoking cessation treatment  Quit attempts | Abstinence  Satisfaction  Usability | SZ | 162  (84/78) | Male (67%)  46 years (11.3) | Community | - | 14.5 (10.5) | Good |
| Ferron  (2011)  United States | Quasi-experimental | Website development | Usability  Acceptability | NR | 71 | Male (63%) | Community | - | - | Poor |
| Ferron  (2017)  United States | Quantitative evaluation | Usability  Usefulness | - | SZ, BPD | 21 | Male (81%) | Community | 7 (2.0) | 26.0 (9.0) | Good |
| Gowarty  (2020)  United States | Qualitative | Perceptions Experience | - | PNS | 22 | Male (54%) | Community | - | - | High |
| Gowarty  (2021)  United States | Prospective | Usability | - | SZ | 17 | Male (86%)  30 years (3.6) | Community | 5.1 (2.1) | 18.5 (8.2) | High |
| Halverson (2022)  United States | RCT – Secondary analysis | Engagement Cognition | - | SZ, MDD, BPD | 62  (33/29) | Male (40%)  46 years (11.1) | Community | 5.0 (2.5) | 17.7 (21.1) | Good |
| Heffner  (2018)  United States | RCT – secondary analysis | Treatment utilisation  Abstinence 30-day PPA |  | BPD, SAFD | 1787 | Male (20%)  46 years (13.3) | Community | 5.6 (2.2) | - | Fair |
| Heffner  (2020)  United States | RCT – Pilot | Feasibility  Acceptability Utilisation, Abstinence | - | BPD | 51 | Male (55%)  49 years (10.8) | Community | 6.2 (2.1) | 19.1 (8.2) | Good |
| Herbst  (2019)  United States | Qualitative | Acceptability, Usefulness, Usability | - | PTSD | 20 | Male (100%)  40 years (15.9) | Community | 4.6 (2.4) | - | High |
| Hicks  (2017)  United States | RCT | Abstinence – biochemically verified 7-dat PPA | Biochemically verified long-term abstinence at 3- and 6 months | PTSD | 11  (5/6) | Female (80%)  53 years (10.5) | Community | 7.2 (0.8) | 17.0 (8.2) | Fair |
| Klein  (2019)  Austraila | Qualitative | User experience | - | SZ, BPD, PD | 12 | Male (67%) | Community | - | 28.0 (9.9) | High |
| Leutwyler (2021)  United States | Quantitative evaluation | Interest in quitting  Change in smoking behaviour | - | SMI | 5 | Male (80%)  47 years (11.7) | Community | - | - | Fair |
| Medenblik (2020)  United States | RCT Pilot | Acceptability Feasibility  Smoking cessation knowledge | - | SZ, PNS | 34  (21/13) | Male (80%)  48 years (9.9) | Community | 5.9 (2.3) | 17.6 (11.7) | Good |
| Minami  (2018)  United States | Open-label, non-randomised feasibility study | Feasibility acceptability | - | MDD | 8 | Female (100%)  55 years (4.6) | Community | 5.2 (1.9) | 12.8 (6.9) | Good |
| Minami  (2021)  United States | RCT – Pilot | Efficacy | - | MDD, BPD | 49  (25/24) | Male (24.5%)  49 years (11.6) | Community | 4.8 (1.7) | 10.5 (4.8) | Good |
| Sharma-Kumar  (2021)  Australia | Quasi-experimental | Acceptability | - | MDD, AD, BPD, SZ, PTSD, DD, PD, PNS | 29 | Male (52%)  43 years | Community | - | 20.0 | High |
| Vilardaga (2016)  United States | Cross-sectional | User experience | - | SZ, BPD, MDD | 5 | Male (100%)  51.2 (4.3) | Community | - | - | High |
| Vilardaga (2018)  United States | Formative evaluation | Usability | - | SMI | 7 | - | Community | - | - | High |
| Vilardaga (2019)  United States | Case study - crossover without repeat measures | Usability  User experience User engagement | - | SZ, PNS, MDD, MD | 7 | Male (43%)  45 years (9.5) | Community | 5.0 | - | Good |
| Vilardaga  (2020)  United States | RCT – Pilot | Feasibility Acceptability, Efficacy | Reduction in tobacco consumption Quit attempts | SZ, MDD, BPD | 62  (33/29) | Male (40%)  46 years (11.1) | Community | 5.0 (2.5) | 17.7 (21.1) | Fair |
| Wilson  (2019)  United States | Cohort | Tailoring of the intervention | - | SZ, PNS | 13 | Male (61%)  48 years (11.0) | Community | 4.9 (1.8) | 15.0 (6.6) | Good |

Diagnosis: AD – Anxiety disorders; BPD – Bipolar disorder; CD – conduct disorder; DD - developmental disorder; ED Eating disorder; Mood disorder – MD; MDD – Major depressive disorder; NC – Neurocognitive disorder; OCD – obsessive-compulsive disorder; PD – Personality disorder; PNS Psychosis not specified; PTSD – Post-traumatic stress disorder; SAFD – Schizoaffective disorder; SZ – schizophrenia, SD – Somatic symptom disorder

FTND - Fagerstrom Test for Nicotine Dependence; MMAT – Mixed Methods Appraisal Tool^28^
