# Supplementary material for: Effectiveness and Experience of Implementing Digital Interventions to Promote Smoking Cessation Among Adults With Severe Mental Illness: A Systematic Review and Meta-analysis
Source: Nicotine Tob Res. 2024 Oct 9;27(6):951–61. doi: 10.1093/ntr/ntae237 (PMC12095810; doi:10.1093/ntr/ntae237)
Supplement: ntae237_suppl_Supplementary_Table_S4 [file ntae237_suppl_supplementary_table_s4.docx]

**Supplementary Table 4. Characteristics of interventions tailored for participants with SMI**

| **Author (Year)** | **Intervention** | **Intervention content** | **Delivery mode** | **Intervention duration** | **Control group** |
| --- | --- | --- | --- | --- | --- |
| Aschbrenner et al. (2018) | Care2Quit | The intervention was modification of an programme originally developed for support persons of smokers in the general population. Topics in the training included rationale for cessation treatment, the role of the support person, education on readiness to quit, supportive behaviours based on the smoker’s readiness to change, and how to reinforce progress made by the smoker. The primary goal of the intervention was for support persons to link their smoker to a free evidence-based online smoking cessation decision aid for smokers with mental illness. The decision aid had been tailored for ease of use for people with SMI and used motivational interviewing techniques. | Single telephone coaching session/online decision tool | 12 weeks | None |
| Brown et al.  (2021) | Helping Hand 3 | The intervention was designed for the purpose of supporting adults with SMI who smoke in smoking cessation. In addition to usual care, Helping Hand 3 included a motivational interviewing-based counselling session, with specific adaptations tailored for patients with SMI. At the end of the session, participants were offered access to free telephone, text-based, and/or web-based cessation counselling offered by a Quitline service provider. | Telephone, text-based, and/or web-based counselling | Up to 5 proactive calls over 5 months, with unlimited inbound calls | Usual hospital care consisting of brief smoking cessation information and advice from their admitting nurse, self-help materials, and an offer of NRT to use during hospitalisation. |
| Browne et al.  (2021) | Learn to Quit | Theory-based smoking cessation app designed for people with SMI based on principles of Acceptance and Commitment Therapy, and psychoeducation on NRT. The intervention is comprised of 28 modules that facilitate learning of smoking cessation content and skills, as well as daily check-ins of mood, smoking urges and cigarette use. | Smartphone app | 2 weeks | App for the general public: QuitGuide is a smartphone application developed by the NCI that includes health information about smoking, tracking tools (e.g., for cravings), and advice for quitting. |
| Brunette et al.  (2011) | Let’s Talk About Smoking | The intervention was an electronic decision support programme designed for people with SMI who smoke and which takes account of cognitive deficits and limited computer experience. It was developed to stimulate motivation to quit smoking with use of evidence-based treatment. Health behaviour change theory informed the content development. The intervention aimed to increase the user’s motivation to quit smoking and to use cessation treatment to do so. A video-recorded narrator, who identifies himself as a former smoker with mental illness, guides users through the intervention and encourages them to quit smoking. | Web-based | Single session | None |
| Brunette et al.  (2018) | Let’s Talk About Smoking |  | Web-based | Single session | Usual care: Prescriber visit for smoking cessation included a visit with participants’ psychiatrist or nurse practitioner to discuss cessation medications and NRT. |
| Brunette et al.  (2019) | Let’s Talk About Smoking |  | Web-based | Single session | Computerised National Cancer Institute (NCI) Patient Education: A reproduced version of the NCI patient educational handout provided static information about smoking-related diseases and smoking cessation treatments. The content was provided to participants via laptop. |
| Brunette et al.  (2020) | Let’s Talk About Smoking |  | Web-based | Single session | Computerised National Cancer Institute (NCI) Patient Education: A reproduced version of the NCI patient educational handout provided static information about smoking-related diseases and smoking cessation treatments. The content was provided to participants via laptop. |
| Gowarty et al.  (2021) | QuitGuide and QuitStart | The National Cancer Institute (NCI) provides two smoking cessation apps based on behavioural change theories and clinical practice guidelines – one designed for adults (QuitGuide) and the other designed for teens (QuitStart) in the general population. Both apps encourage the user to set a quit date within 14 days, provide information about quitting, and allow users to enter personal smoking data. They allow users to track cigarette cravings and provide information regarding coping with these experiences. They also provide information on users’ progress, such as cigarettes avoided, and money saved by not smoking. In addition, users can connect to social media through the apps. | Smartphone app | 2 weeks | None |
| Halverson et al. (2022) | Learn to Quit | Theory-based smoking cessation app designed for people with SMI based on principles of Acceptance and Commitment Therapy, and psychoeducation on NRT. The intervention is comprised of 28 modules that facilitate learning of smoking cessation content and skills, as well as daily check-ins of mood, smoking urges and cigarette use. | Smartphone app | 2 weeks | App for the general public: QuitGuide is a smartphone application developed by the NCI that includes health information about smoking, tracking tools (e.g., for cravings), and advice for quitting. |
| Heffner et al.  (2018) | WebQuit | The intervention is tailored to people with SMI who smoke. It is based on Acceptance and Commitment Therapy, WebQuit has four parts: (1) make a plan, allowing users to develop a personalised quit plan, (2) be aware, containing three exercises to illustrate problems with trying to control thoughts, feelings and physical sensations, (3) be willing, containing eight exercises to help users practice allowing thoughts, feelings and physical sensations that trigger smoking, and (4) be inspired, containing 15 exercises to help participants identify deeply held values inspiring them to quit smoking. | Web-based | Available for login at any time for 12 months | National Cancer Institute’s Smokefree.gov website that includes (1) guidance on setting a quit date, (2) interactive content such as screening questionnaires for depression and nicotine dependence, and (3) information about the health effects of smoking. |
| Heffner et al.  (2020) | WebQuit Plus | WebQuit Plus was built on the foundations of the WebQuit intervention (described above). WebQuit Plus contains an ask-the-expert feature that allows users to post questions and view responses to their own and others’ queries. | Web-based | 10 weeks | National Cancer Institute’s Smokefree.gov website that includes (1) guidance on setting a quit date, (2) interactive content such as screening questionnaires for depression and nicotine dependence, and (3) information about the health effects of smoking. |
| Herbst et al.  (2019) | Stay Quit Coach | App developed to complement an evidence-based integrated care protocol for people with PTSD who smoke. The app provides empirically supported smoking cessation interventions targeting PTSD symptoms associated with smoking lapse. Stay Quit Coach includes controlled breathing and coping plans features. Users can personalise the app with their own reasons for quitting, medication dosing schedule and reminders, timed motivational messages, individualised coping plans, and a ‘money-saved’ calculator. | Smartphone app | 8 weeks | None |
| Hicks et al.  (2017) | Stay Quit Coach | App developed to complement an evidence-based integrated care protocol for people with PTSD who smoke. The app provides empirically supported smoking cessation interventions targeting PTSD symptoms associated with smoking lapse. Stay Quit Coach includes controlled breathing and coping plans features. Users can personalise the app with their own reasons for quitting, medication dosing schedule and reminders, timed motivational messages, individualised coping plans, and a ‘money-saved’ calculator. | Smartphone app | Not specified | Smoking cessation counselling and smoking cessation medications, with a mobile contingency app enabling participants to receive monetary compensation for smoking abstinence. |
| Klein et al.  (2019) | Kick-It | The intervention tailored an existing app used by people in the general population to the needs of people with SMI. An app based on the Theoretical Domains Framework and contains 4 core features: the smoke and crave profile feature which tracks user’s smoking and quitting behaviours in real time and delivers in-time quit strategies; the Kick.it stack feature contains education and strategies to assist app users during their quit attempt; the social network platform is a unique app feature that leverages peer support, and the missions and treatment goals feature that is based on an incentive and reward system that encourages users to engage in daily health-enhancing activities and log their pharmacotherapy use. | Smartphone app | Not specified | None |
| Medenblik et al. (2020) | iCommit | Smoking cessation intervention tailored for people with SMI that combines mobile technology with behavioural, cognitive-behavioural, and pharmacologic approaches. The components of the intervention included the following: 1) behavioural therapy in the form of mobile contingency management (mCM) designed to increase early abstinent rates; 2) pharmacotherapy for smoking cessation (including NRT and bupropion); and 3) five sessions of guideline-based cognitive-behavioural smoking cessation counselling designed to increased coping skills specific to smoking cessation. Participants had the choice as to whether they preferred their first session as a home visit or by telephone. All remaining counselling sessions were conducted by telephone. | Smartphone app and telephone | 6 weeks | The intensive treatment comparison (ITC) included all the iCommit components except for the mobile contingency management. Participants also received five CBT sessions. |
| Minami et al.  (2018) | mSmart Mind | The intervention was designed for people with mood disorders. The app randomly prompts participants 5 times per day (at least 1.5 h apart) during the participant's reported waking hours to complete ecological monetary assessment reports, as well as to engage in mindfulness practice and to complete a post-mindfulness practice report. The app also prompts participants to videotape themselves testing their CO levels twice a day (at the end of the first and last reports of the day) for 14 days following the target quit date. Participants also received counselling sessions via telephone. | Smartphone app and telephone | 4 weeks | Two in-person individual pre-quit counselling sessions and two post-quit sessions. No comparison smartphone intervention was included. |
| Minami et al.  (2021) | mSmart Mind |  | Smartphone app and telephone | 4 weeks | Two in-person individual pre-quit counselling sessions and two post-quit sessions. No comparison smartphone intervention was included. |
| Vilardaga et al. (2016) | QuitPal | QuitPal is a free smoking cessation app developed by the National Cancer Institute for the general population. This app provides tools based on US Clinical Practice Guidelines, which have received extensive empirical support and are considered the gold standard in smoking cessation. QuitPal was iteratively developed using user-centred design principles. | Smartphone app | 3 days | None |
| Vilardaga et al.  (2018) | Learn to Quit | Theory-based smoking cessation app designed for people with SMI based on principles of Acceptance and Commitment Therapy, and psychoeducation on NRT. The intervention is comprised of 28 modules that facilitate learning of smoking cessation content and skills, as well as daily check-ins of mood, smoking urges and cigarette use. Theory-based smoking cessation app designed for people with SMI based on principles of Acceptance and Commitment Therapy, and psychoeducation on NRT. The intervention is comprised of 28 modules that facilitate learning of smoking cessation content and skills, as well as daily check-ins of mood, smoking urges and cigarette use. | Smartphone app | 2 weeks | None |
| Vilardaga et al.  (2019; 2020) | Learn to Quit | Theory-based smoking cessation app designed for the general population but tailored for people with SMI based on principles of Acceptance and Commitment Therapy and psychoeducation on NRT. The intervention is comprised of 28 modules that facilitate learning of smoking cessation content and skills, as well as daily check-ins of mood, smoking urges and cigarette use. | Smartphone app | 2 weeks | App for the general public: QuitGuide is a smartphone application developed by the NCI that includes health information about smoking, tracking tools (e.g., for cravings), and advice for quitting. |
| Wilson et al.  (2019) | iCommit | Intervention tailored to adults with SMI. It included smoking cessation treatment that combined mobile technology with behavioural, cognitive-behavioural, and pharmacologic approaches. The components of the intervention included the following: 1) behavioural therapy in the form of mobile contingency management (mCM) designed to increase early abstinent rates; 2) pharmacotherapy for smoking cessation (including NRT and bupropion); and 3) five sessions of guideline-based cognitive-behavioural smoking cessation counselling designed to increased coping skills specific to smoking cessation. Participants had the choice as to whether they preferred their first session as a home visit or by telephone. All remaining counselling sessions were conducted by telephone. | Smartphone app and telephone | 6 weeks | None |
